# Standardization of Neurotechnology for Brain-Machine Interfacing: State of the Art and Recommendations

**DOI:** 10.1109/OJEMB.2021.3061328

**Published:** 2021-02-23

**Authors:** RICARDO CHAVARRIAGA, CAROLE CARY, JOSE LUIS CONTRERAS-VIDAL, ZACH MCKINNEY, LUIGI BIANCHI

**Affiliations:** ^1^ Chair, IEEE-SA IC Activity - Neurotechnology for Brain-Machine InterfacingZurich University of Applied Sciences, ZHAW 30944 Winterthur Switzerland; ^2^ Chair, IEEE EMB Standards Committee Engineering in Medicine and Biology Society; ^3^ FIEEE, FAIMBE, Co-Chair IEEE SA IC Activity Neurotechnology for Brain-Machine Interfacing, NSF IUCRC BRAINUniversity of Houston; ^4^ Chair, IEEE P2794 Standards Working Group – Reporting Standards for in vivo Neural Interface Research (RSNIR)The BioRobotics Institute; European Ctr of Excellence in Robotics & AI, Scuola Superiore Sant'Anna Pisa Italy; ^5^ Chair, IEEE P2731 Standards Working Group – Unified Terminology for Brain-Computer Interfaces Civil Engineering and Computer Science Engineering”Tor Vergata” University of Rome 9318 Italy

Research and development of brain-machine interfacing (BMI) systems and related neurotechnologies are at a crucial stage in their history. Progress in sensing technologies, advanced materials, robotics and artificial intelligence provides possibilities that until recently were considered science fiction. Direct neural interfacing with external or virtual devices can usher a new era where merging biological and artificial intelligence will have significant impact in multiple domains.

First and foremost, BMIs are becoming powerful tools to improve our understanding of the brain and nervous system. In turn, this can lead to better therapeutic and assistive approaches to tackle healthcare challenges, as well as new modalities for human-machine interaction that may have transformative effects in many consumer-oriented applications.

Not surprisingly, these technologies have generated remarkable interest and investment from both public and private organizations, including several publicly funded national and regional brain initiatives, as well as the worldwide creation of a large number of neurotechnology enterprises. Some projections expect the neurotechnology market to reach a valuation of USD 19 billion by the end of 2026 [1].

Despite their promise, BMI may be on the cusp of the hype curve, facing increasing pressure to demonstrate concrete value to users. In addition to the numerous technical challenges inherent to developing safe, efficacious, and reliable solutions, researchers and developers face the complex human-centered challenges of discerning which data and use cases provide the most value to which users and organizations.

The development and commercialization of BMI systems require researchers, clinicians, manufacturers, and regulatory bodies to ensure that these devices comply with well-defined safety and effectiveness criteria. BMI systems typically require integration of multiple modules comprising measurement and analysis of neural activity, and provision of feedback to the user through various means, such as visual displays, virtual reality systems, haptic interfaces, and exoskeleton. The scarcity of specific BMI and broader neurotechnological standards hinders the design of new devices for interoperability and regulatory compliance, thus posing a barrier to widespread user access (industrial, clinical, and consumer) and potential benefit.

It is thus imperative for the BMI community to have a good understanding of the current state of the standards in the field, as well as the main gaps that need to be addressed. For this reason, the IEEE Standards Association (IEEE-SA), IEEE Engineering in Medicine and Biology Society (EMBS)'s Technical Committee on Standards, and IEEE Brain Initiative initiated an Industry Connections Activity (ICA) on the topic of *Neurotechnologies for Brain-Machine Interfacing* (NT-BMI; IC17-007) [2]. This initiative is dedicated to evaluating existing standards and best practices for BMI system design and usage, as well as to identifying priority areas for new standards. The NT-BMI established a multi-stakeholder group, comprising experts and representatives from academia, industry, and regulatory agencies worldwide. In February 2020, we released an IEEE Standards Roadmap [3] providing a comprehensive overview of the current practices and future requirements for NT-BMI standardization. This activity has also spawned three Standards Working Groups: IEEE P2725.1: Standard for Microwave Structural, Vascular or Functional Medical Imaging Device Safety [4, p. 1], IEEE P2794: Reporting Standards for *in vivo* Neural Interface Research (RSNIR) [5], and IEEE P2731: Standard for a Unified Terminology for Brain-Computer Interfaces [6].

BMI systems typically integrate multiple elements or components, often comprising technologies at different levels of maturity. Available standards may thus vary considerably across constituent elements. Since most BMIs place the ‘user-in-the-loop,’ such standards should address the end user's needs, attention (engagement) and intention, including user instructions. To reflect the nature of BMIs as ‘complex systems of systems,’ the NT-BMI Standards Roadmap is structured in five functional areas identified by the NT-BMI Group: (1) sensor technology, (2) end effectors, (3) data representation, storage & sharing, (4) user needs, and (5) performance assessment & benchmarking. This editorial and accompanying Emerging Topics papers in this journal present and discuss the main findings and recommendations of the NT-BMI and related working groups.

*BMI sensor technologies:* encompass a broad spectrum of transducer types, including both invasive and non-invasive modalities. They range from well-established and widely used techniques such as electroencephalography to emerging approaches like microwave and ultrasound imaging, stentrodes, neural lace, and neural dust.

Among the five functional areas, sensing technologies are arguably the area with the highest level of standardization. Nonetheless, there is no established standard for time synchronization among different systems and modules, since the interfaces and ports to those systems vary widely. The NT-BMI Group also recommends that consumer-grade sensors comply with safety and performance standards consistent with clinical device requirements, given the prevailing trend towards use of consumer device data for health and wellness applications [7].

*End effector systems for BMIs:* include actuators, virtual or physical devices, and feedback mechanisms. They can be broadly categorized into exoskeletal devices, prosthetic devices, virtual/augmented reality interfaces, and neurostimulation devices (peripheral, spinal, transcranial, and intra-cranial).

Priorities for standardization in this functional area include data communication protocols between the end-effector and other BMI elements, shared control strategies and architectures, and unification of terminologies. The first paper of this series, “A Roadmap Towards Standards for Neurally Controlled End Effectors” [8] provides more detailed information on this topic.

*Data Representation, Storage, & Sharing:* There have been a variety of efforts to define data formats for various biosignals used in BMI systems, in the forms of file format specifications, standards, software frameworks, and initiative groups. Nonetheless, efficient data storage and secure interoperability has emerged as the ‘need of the hour’ for standardization – in particular, specific to closed loop applications. Similar to other highly-sensitive-data-based applications, requirements for portability, interoperability, and privacy are essential for viable BMIs and associated systems. To this end, the data standards now being developed by IEEE P2933 WG (“TIPPSS for Clinical IoT”) may provide a useful framework for BMIs [9]. IEEE P2731 WG is also working on defining the information that should be stored into data files to allow automatic processing of BMI signals without the need to access additional resources (e.g., scientific papers or other documents), which is time-consuming and requires human intervention.

*User Needs:* The specification of device users, use cases, and the fulfilment of user needs remain foundations of the user-centered design (UCD) process for both medical and consumer devices. Indeed, UCD processes (including human factors/ usability engineering: HFE/UE) have been shown to yield significant downstream benefits in product development life cycles, including higher user satisfaction, better product adoption, reduced net development costs, and early insight regarding future products and markets [10]. While usability evaluation is a required element of risk management for medical devices and there exist high-level standards defining HFE/UE frameworks [11], [12], the development and maintenance of HFE/UE processes for specific devices remains the resource-intensive responsibility of developers.

To promote the effective, efficient identification and fulfillment of user needs, NT-BMI standardization efforts should thus develop additional HFE/UE standards that complement existing frameworks by defining technology-specific methodologies and quality metrics, in a manner adaptable to different users and use cases [13]. Such standards will improve the rigor of neurotech R&D, the quality of resulting devices, and will reduce the time and resources required for clinical validation and commercialization.

*Specification, Performance Assessment & Benchmarking:* have been identified as additional clear priorities for standardization. Importantly, these protocols and metrics should extend beyond the separate evaluation of individual sub-systems/components and allow assessment of the entire BMI system during closed-loop operation under intended use conditions. The lack of consensus terminology, metrics, and reporting criteria to this end hinder the assessment and comparison of different systems used for related applications. Accordingly, the second paper of the present NT-BMI series formalizes a “Functional Model for Unified Brain-Computer Interface Terminology” [14]. In complement, the third paper in the series presents a set of “Preliminary Minimum Reporting Requirements for *in-vivo* Neural Interface Research” for implantable neural interfaces [15].

By integrating standardized benchmarking protocols and metrics, commonly agreed-upon terminology, and comprehensive scientific reporting guidelines, the NT-BMI initiative seeks to cultivate an ecosystem of increased information interoperability spanning the fields of neuroscience, neurotechnology, and neural rehabilitation. By enabling more rigorous psychometric investigations, this interoperability will in turn promote more robust fulfillment of user needs and better alignment of NT-BMI to serve collective human health and wellbeing. To this end, such technological standards must complement broader initiatives on the ethical and responsible development of technology such as the IEEE NeuroEthics Framework [16], the Ethically Aligned Design guidance [17], and the OECD recommendations for Neurotechnology Enterprises [18].

*General recommendations:* Beyond the specific functional areas, the NT-BMI Standards Roadmap has also distilled the following general recommendation: (1) Efforts should be invested to educate the BMI R&D community on the benefits of standardization with respect to technological design, quality of research, and the ultimate potential for clinical and commercial development. Accordingly, the standards development process should incorporate the perspectives and interests of all neurotechnology stakeholders – including researchers, clinicians, developers, regulatory and scientific reviewers, end users, etc. – via active community engagement by the NT-BMI Group and related initiatives; (2) BMI safety, security and privacy appear as top priorities for standardization. BMI-specific standards in this domain should build on existing principles, standards, and regulatory guidelines for medical and information technologies; (3) Existing efforts to improve scientific reproducibility and open science can be leveraged to establish and consolidate standards for data sharing and reporting on neurotechnology developments; (4) Stakeholders should consider defining complementary and modular standards that promote interoperability, translation, and scaling between consumer and clinical applications; (5) It is important to envision and develop a flexible yet consistent neurotechnology standardization ecosystem that harmonizes community-established best practices, soft law, ethics, international consensus standards, research reporting guidelines, and government regulation. BMI-specific standards should be aligned with existing and emerging standards and regulatory frameworks to address ethical, legal, and societal implications of emerging technologies.

To conclude, it is important to recognize that the scientific and technological foundations for BMI are in perpetual evolution. Hence, the standardization priorities and recommendations identified herein should be re-evaluated through a continual dialog among all stakeholders. The work presented in the NT-BMI Standards Roadmap and this series of papers is thus intended as an invitation to continue this dialog. Those wishing to collaborate or provide feedback are encouraged to contact the corresponding author(s) of interest.


Ricardo Chavarriaga
Chair, IEEE SA IC Activity - Neurotechnologies
for Brain-Machine Interfacing (IC17-007)
Zurich University of Applied Sciences, ZHAW
Winterthur, Switzerland
r_chavarriaga@ieee.org
Carole Carey
Chair, IEEE EMB Standards Committee
Engineering in Medicine and Biology Society
c.carey@ieee.org
Jose Luis Contreras-Vidal
FIEEE, FAIMBE
Co-Chair, IEEE SA IC Activity - Neurotechnologies for Brain-Machine Interfacing (IC17-007)
NSF IUCRC BRAIN
University of Houston, Houston, Texas
jlcontreras-vidal@uh.edu
Zach Mckinney
Chair, IEEE P2794 Standards Working Group – Reporting Standards for in vivo Neural Interface Research (RSNIR)
The BioRobotics Institute; European Ctr of Excellence in Robotics & AI,
Scuola Superiore Sant'Anna, Pisa, Italy
z.mckinney@ieee.org
Luigi Bianchi
Chair, IEEE P2731 Standards Working Group – Unified Terminology for Brain-Computer Interfaces
Civil Engineering and Computer Science Engineering,
“Tor Vergata” University of Rome, Italy
luigi.bianchi@uniroma2.it

